# Effect of Fatty Acid Saturation and Side-Chain Length on the Thermal Behavior and Morphology of Plant Oil-Based Acrylic Polymers

**DOI:** 10.3390/molecules31203650

**Published:** 2026-10-13

**Authors:** Mariia Usiichuk, Bohdan Domnich, Yuliia Romaniuk, Vasylyna Kirianchuk, Andriy Voronov

**Affiliations:** 1 Department of Coatings and Polymeric Materials, North Dakota State University, Fargo, ND 58108, USA; mariia.usiichuk@ndsu.edu (M.U.);; 2 Department of Organic Chemistry, Lviv Polytechnic National University, 79013 Lviv, Ukraine; yuliia.romaniuk@ndsu.edu

**Keywords:** plant oil-based monomers, biobased polymers, fatty acid saturation, side-chain ordering

## Abstract

In this work, saturated plant oil-based acrylic monomers (POBMs) derived from coconut oil (CCM), babassu oil (BBM), and fully hydrogenated soybean oil (HG SBM) were investigated as building blocks for semicrystalline biobased polymers. The effects of fatty acid saturation and side chain length on free radical polymerization, copolymerization, and polymer morphology were evaluated. Increasing fatty acid saturation suppressed allylic chain transfer reactions, resulting in lower chain transfer constants and higher polymer molecular weights than those of POBMs with higher degrees of unsaturation. Copolymerization of CCM with styrene yielded reactivity ratios of r_CCM_ = 0.44 and r_St_ = 0.96 and Alfrey–Price parameters of Q = 0.49 and e = 0.03, demonstrating favorable reactivity of saturated POBMs toward conventional vinyl comonomers in chain copolymerization. Thermal and structural analyses revealed a pronounced dependence of polymer morphology on fatty acid side chain structure. Polymers containing predominantly C12 side chains exhibited limited molecular ordering, whereas highly saturated C16–C18 side chains produced pronounced melting transitions and substantially increased crystallinity. Specifically, semicrystalline morphologies were observed in HG SBM-based (co)polymers with degrees of unsaturation of approximately 4 mol% or lower. Poly(HG SBM) exhibited an enthalpy of fusion of 78 J g^−1^ and degree of crystallinity of 37%, compared with 67 J g^−1^ and 26%, respectively, for poly(HG SBM-*co*-BBM). These findings establish fatty acid saturation and side chain length as complementary molecular design parameters for controlling side chain packing, and morphology in POBM-based polymers. The ability to generate semicrystalline materials from plant oil-derived acrylic monomers provides a strategy for extending the POBM platform toward fully biobased thermoplastic materials.

## 1. Introduction

In our previous work, we developed a one-step transesterification route for converting plant oil triglycerides into plant oil-based acrylic monomers (POBMs) suitable for free radical polymerization [1,2]. The chemical structure of these monomers can be systematically tailored by selecting plant oils with different fatty acid compositions, including soybean, high-oleic soybean, olive, canola, camelina, linseed oils, etc. As a result, the relative contents of saturated, monounsaturated, and polyunsaturated fatty acid moieties incorporated into the POBMs can be varied over a broad range, providing a versatile platform for tuning the physicochemical, thermomechanical, and mechanical properties of the resulting polymers. In particular, the proportion of oleic, linoleic, and α-linolenic acid residues determines the degree of unsaturation of the pendant side chains, which influences polymer flexibility, cross-linking potential, and ultimately material performance [3,4]. In general, POBM homopolymers are highly hydrophobic, possess low glass transition temperatures (*T_g_*) well below ambient temperature, and exhibit relatively low modulus and strength [5].

In contrast, POBMs derived from oils with higher saturation, including babassu, palm, and hydrogenated sunflower oils, produce polymers exhibiting thermal behavior characteristic of semicrystalline materials, with distinct melting transitions [6,7]. Babassu oil contains approximately 90% saturated fatty acids, with lauric acid (C12) accounting for nearly half of its fatty acid composition. This high saturated fatty acid content provides a favorable structural basis for side-chain ordering in the corresponding polymer. Palm oil and hydrogenated sunflower oil are rich in longer saturated palmitic (C16) and stearic (C18) fatty acids, respectively. Their corresponding homopolymers exhibited melting transitions at −13 and −6 °C, respectively, indicating the formation of ordered domains. This behavior is consistent with the well-established tendency of long, linear hydrocarbon chains to organize into ordered crystalline domains, with the resulting crystal structure influenced by chain length [8,9]. Similarly, vinyl polymers bearing linear alkyl side chains containing more than approximately 10–12 carbon atoms are known to undergo side-chain crystallization, resulting in well-defined semicrystalline morphologies [10,11,12]. Such polymers typically exhibit enhanced chemical resistance, reduced flammability, higher modulus, and other attractive properties for a broad range of engineering applications [13,14].

These observations suggest that varying the proportion of highly saturated POBMs in the monomer feed provides an effective strategy for controlling the formation of semicrystalline copolymers and their thermal behavior, offering new opportunities for bioplastics engineering. To further expand the range of monomer compositions, we synthesized an acrylic monomer derived from fully hydrogenated soybean oil (HG SBM), which contains exclusively saturated fatty acid moieties. Based on our previous findings, we hypothesized that copolymerization of HG SBM with coconut oil-based (CCM) or babassu oil-based (BBM) monomers would enable systematic tuning of ordered morphology and thermal transitions, thereby providing a route to biobased materials with properties suitable for bioplastics applications.

## 2. Results and Discussion

To expand the applicability of POBMs to bioplastics engineering, the inherently soft polymers derived from highly unsaturated plant oil-based monomers must be modified to achieve improved thermomechanical performance. We hypothesized that POBMs containing long, saturated fatty acid side chains could promote side-chain crystallization through regular molecular packing, thereby increasing the stiffness and thermal stability of the resulting materials. This approach provides a fully biobased alternative to the incorporation of petroleum-derived comonomers for enhancing polymer performance. However, its successful implementation requires the development of saturated POBMs compatible with free radical copolymerization, together with a fundamental understanding of their homo- and copolymerization behavior and the resulting structure–property relationships.

The present work extends the POBM platform by demonstrating the free radical polymerization and copolymerization of saturated plant oil-based acrylic monomers and by exploiting their ability to form ordered polymer morphologies through side-chain crystallization [15,16,17].

Acrylic monomers derived from coconut, babassu, and fully hydrogenated soybean oils were copolymerized at different feed ratios to systematically vary the degree of saturation and fatty acid composition of the monomer mixture. This molecular design strategy provides a simple means of tailoring the morphology and thermal behavior of POBM-based polymers. Coconut and babassu oils were selected because they are composed predominantly of saturated lauric acid triglycerides but contain small, distinct amounts of unsaturated fatty acids (Table 1). In contrast, fully hydrogenated soybean oil consists almost exclusively of saturated stearic acid triglycerides (Table 1).

Varying the relative proportions of these monomers therefore enabled a systematic investigation of the effects of saturated fatty acid content and chain structure on polymer crystallization and the resulting thermomechanical properties.

### 2.1. Characterisation of Synthesized POBMs

**Figure 1:**
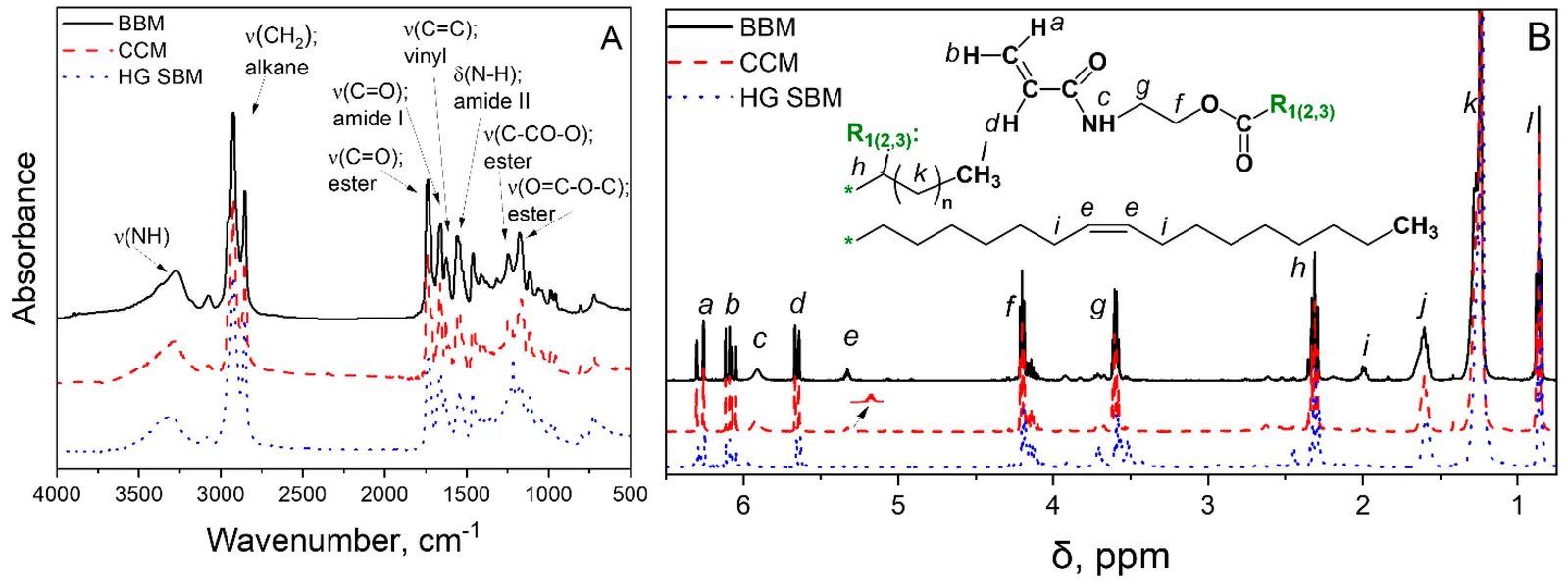
(**A**) FTIR spectra and (**B**) ^1^H NMR spectra of babassu oil- (BBM), coconut oil- (CCM), and hydrogenated soybean oil-based (HG SBM) acrylic monomers. The asterisks (*) indicate the attachment points of the fatty acid chains to the ester groups.

The chemical structures of the coconut oil- (CCM), babassu oil- (BBM), and hydrogenated soybean oil-based (HG SBM) acrylic monomers were confirmed by FTIR spectroscopy (Figure 1A). The broad absorption band at 3200–3400 cm^−1^, assigned to N-H stretching vibrations, together with the characteristic amide I (C=O stretching, 1670 cm^−1^) and amide II (N-H bending, 1540 cm^−1^) bands, confirms the presence of the acrylamide functionality. In addition, the absorption bands at 1740, 1245, and 1180 cm^−1^, corresponding to the ester carbonyl and C-O stretching vibrations, verify the ester functionality of the synthesized monomers.

The ^1^H NMR spectroscopy further confirmed chemical structures of the synthesized monomers (Figure 1B). All three monomers exhibited the characteristic resonances of the acrylic vinyl protons at 6.05–6.65 ppm, the methylene protons adjacent to the amide and ester functionalities at 3.55–4.18 ppm, and the aliphatic protons of the fatty acid chains at 0.85–2.80 ppm. The principal difference among the spectra was observed in the olefinic proton region. The CCM and BBM monomers displayed a resonance 5.30–5.40 ppm, confirming the presence of residual carbon–carbon double bonds in the fatty acid side chains. In contrast, this signal was nearly absent in the spectrum of the hydrogenated soybean oil-based monomer (HG SBM), consistent with its predominantly saturated fatty acid side-chain composition.

The molecular weights of the synthesized monomers were determined by ESI-MS (Figures S4–S6) from the corresponding sodium adducts ([M + Na]^+^). The major peaks for CCM and BBM correspond to a molecular weight of 297 g/mol, consistent with the high content of lauric acid (C12:0) in coconut and babassu oils. In contrast, HG SBM exhibited a molecular weight of 381 g/mol, which agrees with the predominance of stearic acid (C18:0) in fully hydrogenated soybean oil.

The degree of unsaturation of the synthesized monomers was further evaluated by iodine value measurements. As expected, the hydrogenated soybean oil-based monomer (HG SBM) exhibited the lowest iodine value (29 ± 1 g I_2_/100 g), consistent with the nearly complete hydrogenation of the parent soybean oil (iodine value = 3 ± 0.2 g I_2_/100 g). In contrast, the babassu oil- (BBM) and coconut oil-based (CCM) monomers exhibited higher iodine values of 67.5 ± 0.5 and 65 ± 1 g I_2_/100 g, respectively, reflecting the presence of residual unsaturation introduced by the corresponding plant oils.

Taken together, the FTIR, ^1^H NMR, ESI-MS and iodine value analyses confirm the successful synthesis of the target monomers and clearly distinguish HG SBM from CCM and BBM by its saturated, predominantly C18 fatty acid side chains. These structural differences are expected to influence polymerization kinetics, chain packing, ordered morphology, and the thermal behavior of the resulting homo- and copolymers, as discussed in the following sections.

### 2.2. Effect of Fatty Acid Saturation on Free Radical Polymerization of POBMs

According to classical free radical polymerization theory (Equation (1)), the polymerization rate (*R_p_*) is directly proportional to the monomer concentration and to the square root of the initiator concentration [22,23,24]. The characteristic half-order dependence on initiator concentration arises when chain termination occurs predominantly through bimolecular radical combination or disproportionation. Accordingly, deviations from this theoretical relationship provide evidence for additional kinetic processes, such as chain transfer reactions, that alter radical concentrations and influence the overall polymerization behavior.

(1) $$R_{p}=k_{p}{\left(\frac{fk_{d}}{k_{t}}\right)}^{0.5}\left[M\right]{\left[I\right]}^{0.50}$$

where *R_p_* is the polymerization rate, *k_p_* is the propagation rate constant, *f* is the initiator efficiency, *k_d_* is the initiator decomposition rate constant, *k_t_* is the bimolecular termination rate constant, [*M*] is the monomer concentration, and [*I*] is the initiator concentration.

**Figure 2:**
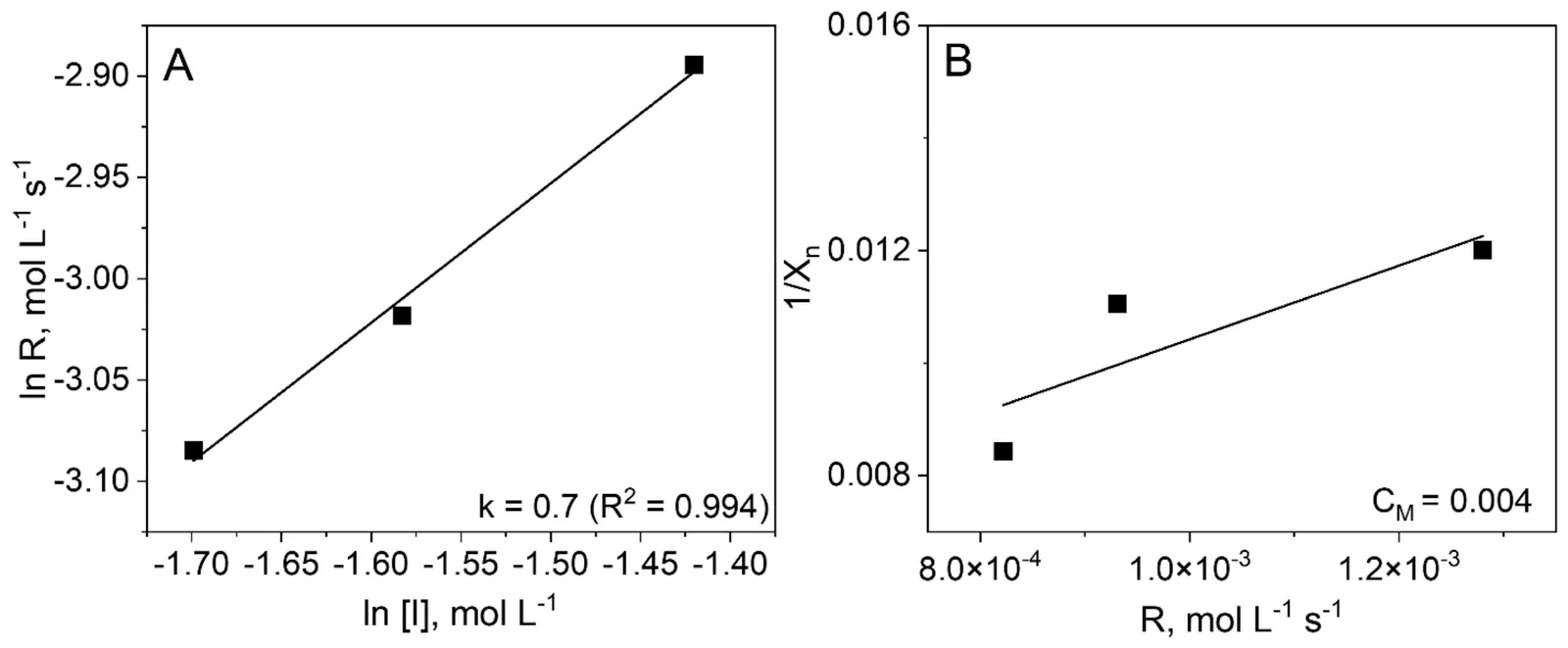
(**A**) Polymerization rate as a function of initiator concentration for the synthesized POBMs at 70 °C. (**B**) Chain transfer constants determined by the Mayo method.

The kinetic analysis was therefore used to evaluate the influence of fatty acid saturation on the free radical polymerization mechanism of saturated POBMs. For this, homopolymerizations of CCM were performed at several initiator concentrations. For each experiment, the polymerization rate (*Rp*, mol L^−1^ s^−1^) was determined from the linear portion of the conversion–time curves at approximately 10–15% monomer conversion, where the reaction rate remained effectively constant (Figure S7). The logarithm of the polymerization rate was then plotted as a function of the logarithm of the initiator concentration, and the reaction order with respect to the initiator was obtained from the slope of the resulting linear relationship (Figure 2A).

The reaction order with respect to the initiator was determined to be 0.7 for CCM, while a value of 0.8 for BBM was reported previously [6]. These values are consistent with our earlier studies demonstrating that the initiator reaction order is strongly influenced by the chemical structure of the POBMs [25]. In particular, decreasing the degree of fatty acid unsaturation shifts the reaction order toward the theoretical value expected for conventional free radical polymerization. This trend is attributed to the lower concentration of allylic carbon–carbon double bonds, which suppresses allylic chain transfer reactions and reduces their contribution to the overall polymerization kinetics.

The contribution of chain-transfer reactions was further quantified using the Mayo method (Figure 2B). The resulting chain transfer constant (*C_M_*) was strongly dependent on the chemical structure of the plant oil-based monomers, particularly the degree of fatty acid unsaturation. As demonstrated in our previous studies, monomers derived from highly unsaturated plant oils exhibited relatively high chain transfer constants (*C_M_* = 0.015–0.033), reflecting the susceptibility of allylic hydrogen atoms to radical abstraction. In contrast, the saturated monomers investigated in the present work exhibited substantially lower *C_M_* values. The chain transfer constant of the coconut oil-based monomer (CCM, *C_M_* = 0.004) was substantially lower than that previously reported for the babassu oil-based monomer (BBM, *C_M_* = 0.013) [6]. This trend is consistent with the lower residual unsaturation of CCM compared with BBM and indicates that reducing the number of allylic hydrogen atoms effectively suppresses chain-transfer reactions during polymerization.

### 2.3. Free Radical Copolymerization of CCM with Styrene

**Figure 3:**
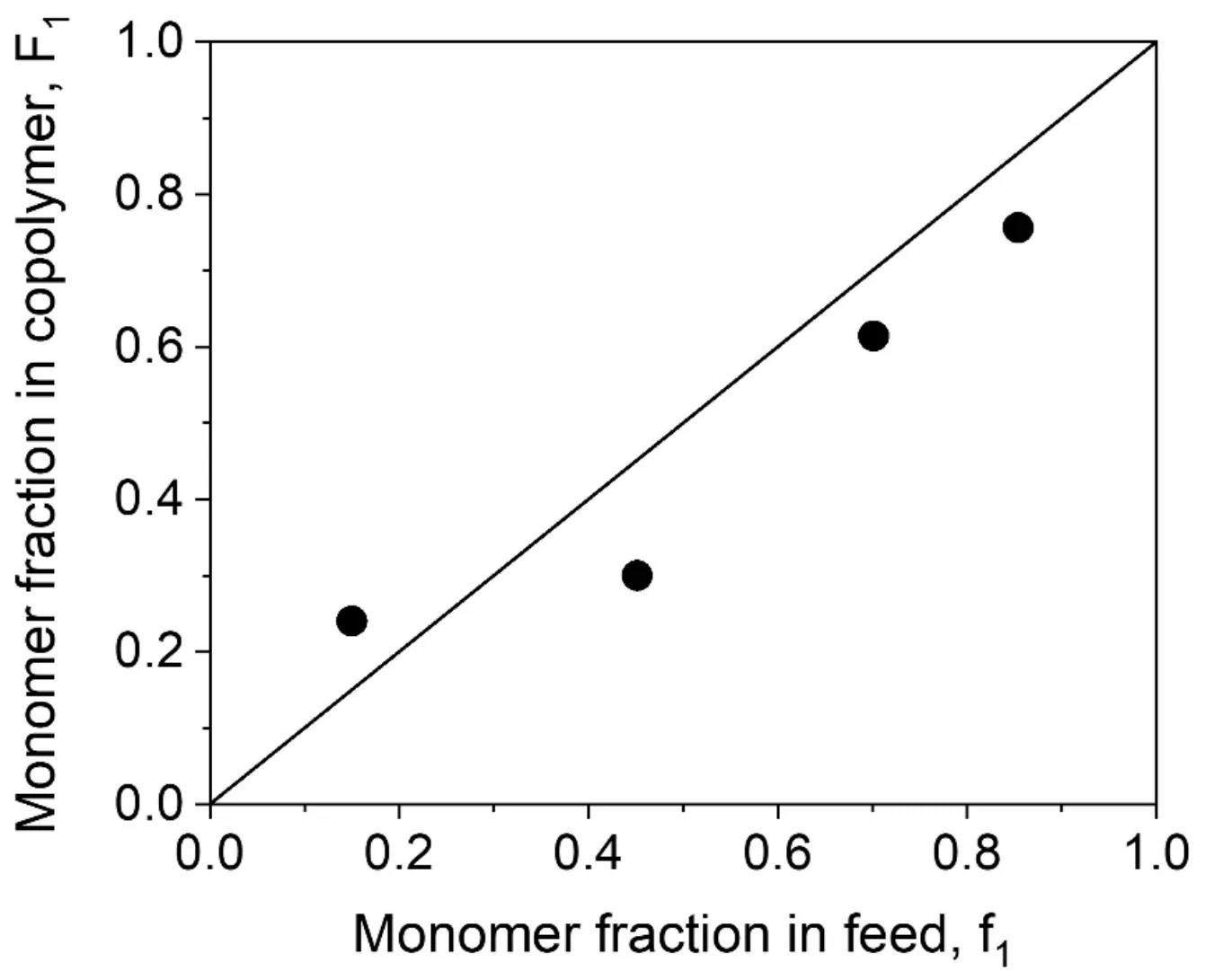
Experimental CCM content in copolymers with styrene as a function of the CCM content in the initial monomer feed.

To further evaluate the potential of saturated POBMs as comonomers for advanced polymeric materials, the coconut oil-based monomer (CCM) was utilized as a representative system and copolymerized with styrene as a model vinyl monomer. Monomer reactivity ratios were determined to quantify the relative reactivities of the two monomers and assess the copolymerization behavior of CCM. Copolymerizations were therefore conducted at low conversion (10–15%) to minimize compositional drift, and the instantaneous copolymer compositions were determined by ^1^H NMR spectroscopy. The monomer feed ratio, *f_i_* = [*m*_1_]*_i_*/[*m*_2_]*_i_*, and the corresponding copolymer composition ratio, *F_i_* = [*M*_1_]*_i_*/[*M*_2_]*_i_*, where *m* and *M* denote the monomer and copolymer compositions, respectively, were analyzed using the Fineman–Ross linearization of the Mayo–Lewis copolymerization equation (Figure 3) [26]. The resulting monomer reactivity ratios, *r*_CCM_ = 0.44 and *r*_St_ = 0.96, indicate that cross-propagation is favored over self-propagation, with CCM-derived radicals exhibiting a greater tendency to add styrene than to undergo homopropagation.

Using the experimentally determined reactivity ratios for the CCM/styrene system and the literature *Q* (*Q* = 1.0) and *e* (*e* = −0.80) parameters of styrene, the Alfrey–Price equation (Equations (2) and (3)) was applied to calculate the corresponding *Q* and *e* values for CCM.

*r*_1_ = *Q*_1_/*Q*_2_ *exp* {−*e*_1_(*e*_1_ − *e*_2_)} (2)

*r*_2_ = *Q*_2_/*Q*_1_ *exp* {−*e*_2_(*e*_2_ − *e*_1_)} (3)

where *r*_1_ and *r*_2_ are the reactivity ratios, *Q*_1_ and *Q*_2_ are the resonance parameters, and *e*_1_ and *e*_2_ are the polar parameters of the corresponding monomers.

The calculated Alfrey–Price parameters (*Q* = 0.49, *e* = 0.03) indicate that CCM possesses moderate resonance stabilization and essentially neutral polar character. Comparison with our previous studies on more highly unsaturated POBMs shows that the *Q* values remain within a narrow range (0.41–0.51), suggesting that resonance stabilization is largely independent of the fatty acid composition of the parent plant oil. In contrast, the *e* parameter is modestly influenced by the degree of fatty acid saturation. The lower *e* value of CCM relative to previously investigated unsaturated POBMs (*e* = 0.09–0.28) indicates a slight reduction in the polar contribution to propagation. However, because all *e* values are close to zero, polar effects are expected to play only a minor role in the copolymerization behavior of plant oil-based acrylic monomers. These results demonstrate that the degree of fatty acid saturation has little influence on the intrinsic copolymerization reactivity of POBMs and that saturation can be varied to tailor polymer morphology without substantially altering the copolymerization characteristics of the acrylic functionality. CCM is readily copolymerizable with styrene, suggesting broad compatibility with conventional vinyl monomers.

### 2.4. Thermal Properties and Morphology of Saturated POBM-Based (Co)polymers

Having demonstrated the favorable free radical polymerization and copolymerization behavior of the saturated POBMs, a series of homo- and copolymers (Table 2) with systematically varied compositions was prepared by bulk polymerization. The objective was to elucidate how the degree of fatty acid saturation influences side-chain ordering, polymer morphology, and the thermal properties of the resulting materials.

The chemical structures and compositions of the resulting polymers were characterized by ^1^H NMR spectroscopy (Figures S8–S12). The complete disappearance of the acrylic vinyl proton resonances at δ 6.05–6.65 ppm confirmed consumption of the polymerizable C=C bonds during polymerization. Copolymer compositions were determined from the ^1^H NMR spectra by comparing the normalized integrals of the residual olefinic protons in the fatty acid side chains with those of the corresponding homopolymers. All spectra were normalized to the terminal methyl protons of the fatty acid side chains (δ ≈ 0.88 ppm, 3H), which are present in each POBM repeat unit and are independent of the degree of fatty acid unsaturation. Following normalization, the integral of the residual olefinic proton resonance at δ ≈ 5.30–5.40 ppm was related to the relative fractions of the corresponding POBM repeat units in the copolymer according to:

(4) $$I_{\mathrm{copolymer}\;}=\;{x\times I}_{{\mathrm{pPOBM}}_{1}\;}+(1-x)\times I_{{\mathrm{pPOBM}}_{2}\;}$$

where *I* is the normalized integral of olefinic proton resonance and *x* is the mole fraction of POBM repeat units in the copolymer.

As shown by ^1^H NMR spectroscopy (Figures S8–S12), the majority of the carbon–carbon double bonds in the POBM fatty acid side chains were retained during polymerization, with the resulting polymers exhibiting unsaturation levels comparable to those of the corresponding precursor oils. This indicates that the degree of unsaturation is largely preserved during POBM synthesis and is therefore reflected in the fatty acid side chains of the resulting polymers. Accordingly, the average degree of unsaturation of each homopolymer was assigned based on the unsaturation of the corresponding precursor oil/POBM. For the copolymers, the average degree of unsaturation was calculated from the experimentally determined copolymer compositions (Table 2) as the mole-fraction-weighted contribution of each POBM component:

*U* = (*y*_1_ × *U*_1_) + (*y*_2_ × *U*_2_) (5)

where *U*_1_ and *U*_2_ are the degrees of unsaturation of the respective POBMs, and *y*_1_ and *y*_2_ are their corresponding mole fractions in the copolymer.

Based on these calculations, the degree of unsaturation of the (co)polymers ranged from 2.2 to 12 mol%, with poly(BBM) exhibiting the highest degree and poly(HG SBM) the lowest. In addition to the degree of unsaturation, the polymers differed in the predominant length of their fatty acid side chains. Poly(BBM), poly(BBM-*co*-CCM), and poly(CCM) contained predominantly C12 fatty acid residues, whereas poly(HG SBM-*co*-BBM) and poly(HG SBM) were enriched in longer C18 residues. These structural differences are expected to affect polymer morphology by side-chain packing and molecular ordering. Thus, this polymer series enables systematic evaluation of the combined effects of fatty acid unsaturation and side-chain length on the morphology and thermal behavior of POBM-based polymers.

The molecular weights and dispersities of the resulting polymers varied systematically with composition (Figure S13, Table 2). Polymers containing higher proportions of unsaturated fatty acid residues exhibited lower molecular weights and broader molecular weight distributions than those enriched in saturated POBM units. This trend is consistent with the greater contribution of chain transfer reactions associated with unsaturated fatty acid moieties. Allylic sites provide additional pathways for radical transfer, thereby limiting chain growth and broadening the molecular weight distribution. Nevertheless, all polymers exhibited number-average molecular weights (*M*_n_) ≥ 40,000 g mol^−1^, demonstrating that bulk polymerization produced relatively high-molecular-weight materials across the entire compositional range.

Although differences in molecular weight may affect chain mobility, packing, and molecular ordering, their contribution is expected to be less pronounced than that of fatty acid side-chain structure within the molecular weight range investigated. Previous studies of comb-like polymers have similarly shown that the dependence of side-chain crystallization on backbone molecular weight diminishes above a threshold molecular weight. For example, Pei et al. reported that the enthalpy of fusion of poly(mono/diethylene glycol *n*-tetradecyl ether vinyl ethers) depended on *M*_n_ at relatively low molecular weights, whereas the melting temperature was only weakly affected [27]. At *M*_n_ > 20,000 g mol^−1^, the enthalpy of fusion approached a nearly constant value, indicating a substantially reduced dependence of side-chain crystallization on backbone molecular weight.

**Figure 4:**
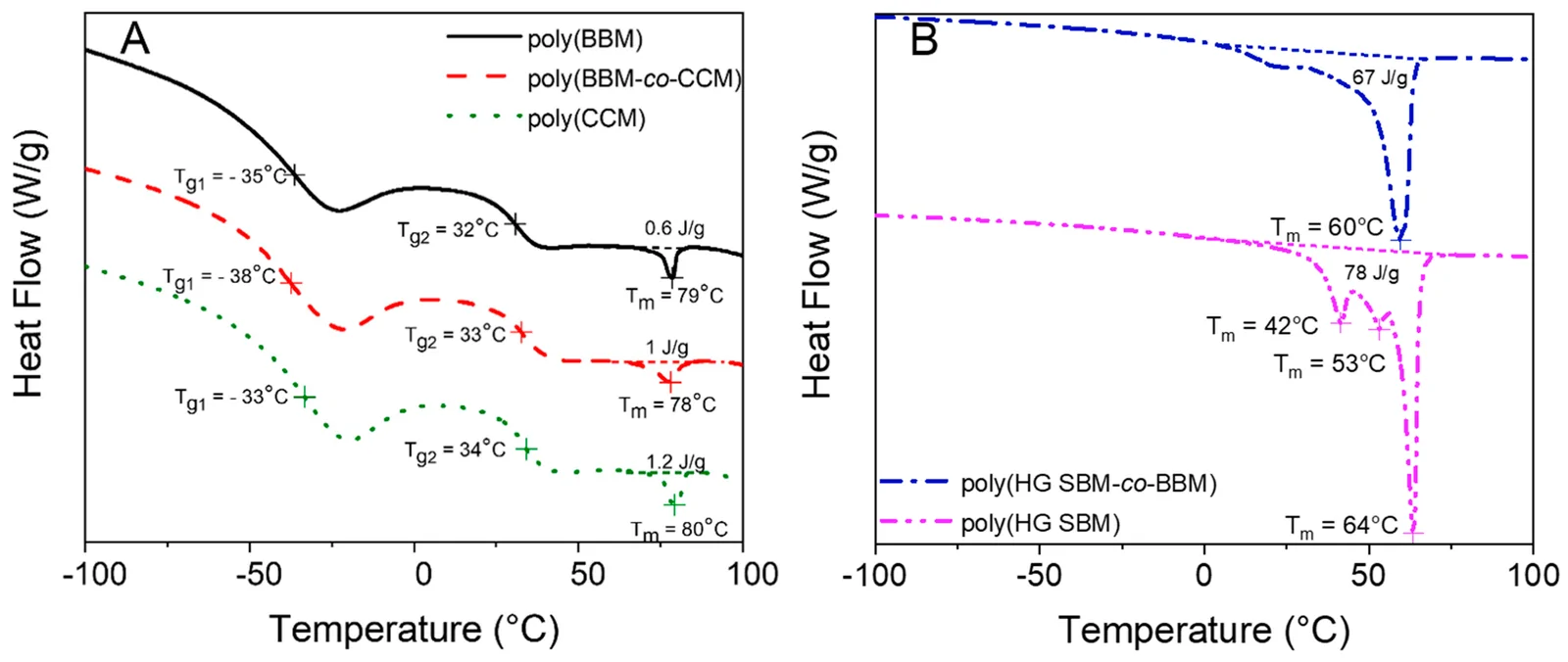
DSC thermograms of (**A**) poly(BBM), poly(BBM-*co*-CCM), and poly(CCM) and (**B**) poly(HG SBM), poly(HG SBM-*co*-BBM).

Conventional differential scanning calorimetry (DSC) was performed using a heat–cool–heat cycle to evaluate the thermal transitions and melting behavior of the polymers (Figure S14). The second heating cycle (Figure 4) was used for analysis to minimize the effects of prior thermal and processing history and enable consistent comparison among the samples. As shown in Figure 4A, the second-heating thermograms of the more unsaturated (co)polymers exhibited two distinct glass transitions at approximately −35 and 33 °C, followed by a melting endotherm at approximately 79 °C. Multiple glass transitions have previously been reported for polymers bearing long side chains and have been attributed to differences in the segmental mobility of the side chains and polymer backbone [28]. The *T_g_*_1_ and *T_g_*_2_ values of poly(BBM), poly(BBM-*co*-CCM), and poly(CCM) were similar, consistent with their predominantly C12 fatty acid side chains and comparable molecular weight characteristics. In contrast, the enthalpy of fusion varied among these polymers, with poly(CCM), which exhibited the lowest degree of unsaturation within this series, showing the highest value (Δ*H*_m_ = 1.2 J g^−1^). The higher enthalpy of fusion indicates a greater extent of morphological ordering, consistent with reduced disruption of side-chain packing by unsaturated fatty acid moieties. Collectively, these results suggest that, for POBM-based polymers containing predominantly C12 fatty acid side chains, decreasing unsaturation facilitates side-chain ordering and more ordered morphologies. However, the relatively low enthalpies of fusion indicate a limited degree of such domains, suggesting that fatty acid side-chain length remains an important structural parameter governing the development of more ordered morphology.

To further evaluate the effect of fatty acid side-chain length, the thermal behavior of polymers enriched in longer C16–C18 saturated fatty acid residues was examined (Figure 4B). In contrast to the predominantly C12-based polymers, the HG SBM-containing polymers exhibited substantially higher enthalpies of fusion and more pronounced melting transitions. Poly(HG SBM), composed predominantly of C16:0 and C18:0 fatty acid residues with only a minor fraction (~2%) of C18:1 residues, exhibited multiple melting endotherms at approximately 42, 53, and 64 °C, with a total enthalpy of fusion (Δ*H_m_*) of 78 J g^−1^. The multiple melting transitions indicate the presence of crystalline domains with different thermal stabilities, potentially arising from variations in side-chain length and packing associated with the C16 and C18 fatty acid residues. The minor C18:1 fraction may further disrupt regular side-chain packing and contribute to heterogeneity within the crystalline domains. In comparison, poly(HG SBM-*co*-BBM), which incorporates shorter C12 side chains from BBM and exhibits a higher degree of unsaturation (~4%), showed a lower Δ*H_m_* of 67 J g^−1^. This indicates that incorporation of shorter and more unsaturated fatty acid side chains disrupts the regular packing of the longer saturated C16–C18 side chains, thereby reducing the extent of morphological ordering.

Modulated differential scanning calorimetry (MDSC) was performed as a complementary analysis on selected samples, including poly(BBM), poly(HGSBM-*co*-BBM), and poly(HGSBM) (Figure S15). MDSC separates the total heat flow into reversing and nonreversing components, enabling heat-capacity-related transitions to be distinguished from kinetic and structural processes. The MDSC analysis confirmed two distinct glass transitions in poly(BBM), consistent with the conventional DSC results. Enthalpic recovery was observed near both transitions, indicating structural relaxation associated with each glass transition process (Figure S15A). In contrast, poly(HGSBM-*co*-BBM) and poly(HGSBM) exhibited pronounced melting transitions without clearly detectable glass transitions (Figure S15B,C). The absence of distinct glass transition events, together with the pronounced melting behavior, is consistent with the substantially greater degree of side-chain packing and crystalline domain formation in these polymers.

Overall, thermal analysis demonstrates that both the degree of fatty acid saturation and side-chain length govern the thermal behavior of POBM-based polymers. Polymers containing predominantly C12 fatty acid side chains exhibited limited crystallization, with increasing saturation producing only a modest increase in the enthalpy of fusion. In contrast, incorporation of longer, highly saturated C16–C18 side chains resulted in substantially higher enthalpies of fusion and more pronounced melting transitions, indicating more extensive crystalline domain formation. These findings suggest that side-chain length plays a particularly important role in promoting crystallization, while the degree of unsaturation modulates the efficiency of side-chain packing and ordering.

**Figure 5:**
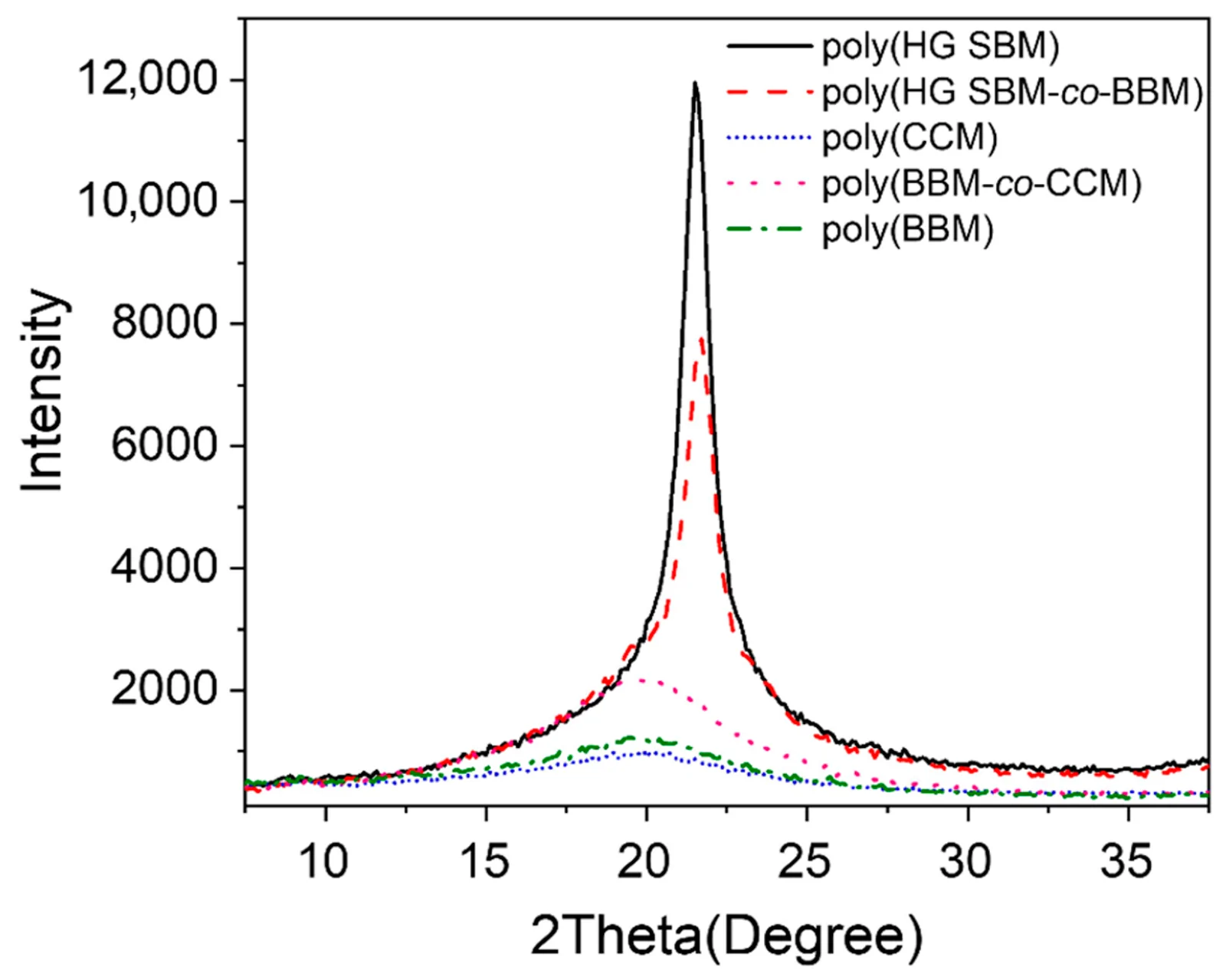
X-ray diffraction patterns of POBM-based (co)polymers.

To further evaluate the crystalline ordering suggested by the DSC results, X-ray diffraction (XRD) analysis was performed on the polymer series (Figure 5). The predominantly C12-based polymers exhibited broad amorphous halos without distinct diffraction peaks, indicating a low degree of crystallinity. In contrast, poly(HG SBM) and poly(HG SBM-*co*-BBM) exhibited distinct crystalline reflections, with calculated degrees of crystallinity of 37% and 26%, respectively. The higher crystallinity of poly(HG SBM) is consistent with its greater enthalpy of fusion and pronounced melting transitions observed by DSC, further supporting the formation of more extensive crystalline domains. The lower crystallinity of poly(HG SBM-*co*-BBM) indicates that incorporation of shorter C12 and more unsaturated fatty acid side chains disrupts the regular packing of the longer saturated C16–C18 side chains.

Overall, the XRD results corroborate the DSC analysis and demonstrate that longer, highly saturated fatty acid side chains promote efficient side-chain packing and the development of semicrystalline morphologies in POBM-based polymers. Within the compositions investigated in this study, reducing the degree of unsaturation to approximately 4 mol% or below, combined with longer fatty acid side chains, provided sufficient structural regularity to promote side-chain ordering and the formation of semicrystalline POBM-based polymers.

## 3. Experimental Section

### 3.1. Materials

The following materials were used in this study: coconut oil (Viva Naturals, New York, NY, USA), babassu oil (VELONA, Mount Prospect, IL, USA), hydrogenated soybean oil (Koster Keunen, Watertown, CT, USA), N-(hydroxyethyl) acrylamide (stabilized with MEHQ, >98%) (TCI America, Portland, OR, USA, CAS 7646-67-5), magnesium sulfate (anhydrous) (VWR Chemicals, Solon, OH, USA, CAS 7487-88-9), potassium hydroxide (Sigma-Aldrich, Saint Louis, MO, USA, CAS 1310-58-3), sodium chloride (EMD Chemicals Inc., Gibbstown, NJ, USA, CAS 7647-15-5), azobisisobutyronitrile (AIBN, Sigma-Aldrich, St. Louis, MO, USA, CAS 78-67-1), styrene (Sigma-Aldrich, St. Louis, MO, USA, CAS 100-42-5), chloroform d (>99.8% + 0.03% TMS) (Oakwood Chemical, Estill, SC, USA, CAS 865-49-6), tetrahydrofuran (VWR Chemicals BDH, Radnor, PA, USA, CAS 109-99-9), *n*-hexane (Sigma-Aldrich, St. Louis, MO, USA, CAS 110-54-3), toluene (Sigma-Aldrich, St. Louis, MO, USA, CAS 108-88-3), and methanol (Sigma-Aldrich, St. Louis, MO, USA, CAS 67-56-1). All solvents were of reagent grade or higher and were used as received.

### 3.2. Synthesis of POBMs

To synthesize POBMs, 115 g of N-(hydroxyethyl) acrylamide was added to 150 g of oil (with acrylamide alcohol to triglyceride molar ratio of 5.9 to 1), 150 mL of tetrahydrofuran, and 0.1 g of 2,6-di-tert-butyl-p-cresol in a 500 mL round-bottom flask equipped with a magnetic stirrer. The mixture was heated to 40 °C (hydrogenated soybean oil) and 50 °C, and 1.5 g of finely ground potassium hydroxide was added slowly with continuous stirring. The reaction was maintained at 40 °C for 3 h (complete homogenization) and then left to stand overnight at room temperature. The resulting product was diluted with 100 mL hexane, washed with 5% brine, treated with magnesium sulfate, and dried under vacuum (80–90% of the theoretical yield).

### 3.3. Solution Polymerization of Coconut Oil-Based Monomer

Coconut oil-based monomer (1.0 mol L^−1^) and AIBN (0.020, 0.025, or 0.038 mol L^−1^) were dissolved in toluene under stirring. The reaction mixture was purged with nitrogen at room temperature for 30 min and subsequently heated at 70 °C for 8 h under a nitrogen atmosphere. Small samples of the reaction mixture were taken to monitor the progress of the reaction using a gravimetric method after precipitation of the polymer into a large excess of methanol and purified by repeated precipitation from toluene into methanol. The purified polymer was then dried under reduced pressure at room temperature to constant weight.

### 3.4. Copolymerization of Coconut Oil-Based Monomer and Styrene

Coconut oil-based monomer and styrene (15:85, 45:55, 70:30, and 85:15 mole parts, respectively) were combined with AIBN (0.038 mol L^−1^) and dissolved in toluene under stirring. The total monomer concentration was maintained at 1.0 mol L^−1^. The reaction mixture was purged with nitrogen (10 min) and subsequently maintained at 70 °C under a nitrogen atmosphere for 6 h, until a total monomer conversion of 10–15% was reached. Aliquots (0.6–0.7 g) of the reaction mixture were collected to monitor the progress of copolymerization. The samples were precipitated in a large excess of methanol, and the conversion was determined gravimetrically. The resulting copolymers were purified by repeated precipitation from toluene into methanol and dried under a nitrogen atmosphere at room temperature to constant weight.

### 3.5. Bulk (Co)polymerization of POBMs

Bulk (co)polymerization of the POBMs was performed by mixing the required amounts of monomers with AIBN (1.5 wt% relative to the total monomer mass). The mixture was heated to 40 °C and stirred for 20 min under a nitrogen atmosphere to ensure homogenization. The temperature was then increased to 70 °C, and the mixture was continuously stirred using a mechanical stirrer for 8 h under nitrogen. After polymerization, the resulting polymer was dissolved in toluene to obtain a 50 wt% solution and purified by repeated precipitation from toluene into methanol. The purified polymers were dried under a nitrogen atmosphere at room temperature to constant weight.

### 3.6. Characterization of Synthesized Materials

To determine (co)polymer yield, approximately 1 mL of the (co)polymer solution was precipitated in 6 mL of methanol to remove unreacted monomers. The precipitation was repeated at least three times to ensure complete monomer removal. The purified copolymer was then dried in an oven at 100 °C for 5 h.

(6) $$\mathrm{Yield}\;\left(\%\right)=\left(\;\frac{\left(\frac{W_{4}}{W_{5}}\right)\times 100}{\mathrm{Solid}\;\mathrm{content}\;(\%)}\right)\times 100$$

where W_4_ is the weight of the dried, purified polymer sample, and W_5_ is the weight of the (co)polymer solution used.

Proton nuclear magnetic resonance (^1^H NMR) spectroscopy was performed on Avance III HD 400 high-performance digital NMR spectrometer (Bruker, Billerica, MA, USA) using 32 scans and a relaxation time of 4 s. Samples were prepared by dissolving 0.03 g of material in 0.6 mL of CDCl_3_.

Fourier transform infrared (FTIR) spectra were recorded using a Nicolet 8700 FTIR spectrometer (Thermo Scientific, Waltham, MA, USA) with KBr optical discs (Alfa Aesar, Ward Hill, MA, USA). POBMs (0.3 g) were dissolved in 3 mL of acetone or chloroform, applied onto the KBr discs, and dried at room temperature prior to analysis.

High-resolution mass spectra were acquired using a SYNAPT G2-Si mass spectrometer (Waters Inc., Milford, MA, USA) equipped with an electrospray ionization (ESI) source. Samples were dissolved in methanol, and sodium adducts ([M + Na]^+^) were monitored in positive-ion mode.

Iodine value (IV) was determined according to the Wijs method. A 0.1 g monomer sample was dissolved in 20 mL of carbon tetrachloride (CCl_4_) in a 500 mL glass flask. Subsequently, 25 mL of Wijs solution was added, and the mixture was stored in the dark at 20 °C for 1 h. After incubation, 30 mL of 0.1 N KI solution and 100 mL of recently boiled and cooled deionized water were added. The solution was titrated with 0.1 N sodium thiosulfate solution under constant stirring until the yellow color nearly disappeared. Starch indicator (1–2 mL) was then added, and the titration continued until the blue color disappeared. A blank determination was carried out under identical conditions. Blank and sample determinations were each performed in triplicate. The iodine value was expressed as grams of iodine absorbed per 100 g of sample and calculated using the following equation:

*IV* = [(*B* − *S*) × *N* × 12.69]/*m_sample_* (7)

where B is the titration of the blank in milliliters, S is the titration of the sample in milliliters, and N is the normality of the sodium thiosulfate solution.

Gel permeation chromatography (GPC) was performed using a Tosoh Bioscience EcoSEC HLC-8320 system (Tokyo, Japan) equipped with a Waters 1515 high-performance liquid chromatography pump (Milford, MA, USA), a Waters 2410 refractive index detector (Milford, MA, USA), and two 10 mm PL-gel mixed-B columns (porous polystyrene/divinylbenzene cross-linked matrix). Tetrahydrofuran (THF) was used as the eluent at a flow rate of 0.35 mL/min, with the column temperature maintained at 40 °C. Calibration was performed using EasiVial polystyrene standards (Agilent Technologies, Santa Clara, CA, USA). For sample preparation, 0.015 g of polymer was dissolved in 3 mL of THF and filtered through a 0.22 μm poly(vinylidene fluoride) membrane filter prior to injection.

Differential scanning calorimetry (DSC) was performed using a TA Instruments DSC 2500 calorimeter (New Castle, DE, USA). Samples (5–10 mg) were sealed in aluminum crucibles. Conventional DSC measurements were conducted using a heat–cool–heat cycle, with the samples initially heated from room temperature to 100 °C, cooled from 100 to −120 °C, and subsequently reheated to 100 °C at a rate of 10 °C min^−1^. Measurements were performed under a dry nitrogen atmosphere at a flow rate of 50 mL min^−1^. Modulated DSC (MDSC) measurements were conducted from −70 to 100 °C at a heating rate of 2 °C min^−1^, using a modulation period of 60 s and a temperature amplitude of ±1 °C under the same nitrogen atmosphere.

X-ray diffraction (XRD) analysis was conducted on films by using Rigaku Ultima IV Versatile X-Ray diffractometer (Tokyo, Japan) using X-ray source of Cu Kα (λ = 1.54 Å) radiation generated at a voltage of 40 kV and current of 40 mA. The diffraction angle 2θ was scanned from 3° to 40° at a scanning rate of 3°/min. X-ray diffraction patterns were obtained at room temperature. Poly(BBM), poly(CCM), and poly(BBM-*co*-CCM) samples were analyzed as films cast from toluene solutions, dried at room temperature, with a thickness of approximately 70 ± 3 μm. Poly(HG SBM) and poly(HG SBM-*co*-BBM) samples were analyzed in powder form. The degree of crystallinity was calculated from the crystalline and amorphous contributions to the diffraction pattern using:

(8) $$X_{c}=\;\frac{A_{c}}{A_{t}}\times 100\%\;$$

where *A_c_* and *A_t_* are the integrated area of the crystalline reflection and the total integrated area under the entire diffraction pattern (crystalline reflection + amorphous halo), respectively, determined using Origin 2018 version 9.5 by OriginLab Corporation.

## 4. Conclusions

Plant oil-based acrylic monomers (POBMs) provide a renewable platform for designing polymers with properties controlled by the fatty acid triglyceride composition (unsaturation and saturation degrees) of the parent oils. In our previous work, the degree of fatty acid unsaturation was shown to directly influence free radical polymerization behavior, properties and performance of POBM-based polymers.

This study establishes saturated POBMs as versatile renewable building blocks for the preparation of semicrystalline polymers with tunable thermal and morphological properties. Increasing saturation suppressed allylic chain transfer reactions, resulting in lower chain transfer constants and facilitating the formation of higher-molecular-weight polymers in comparison to more unsaturated POBMs. Copolymerization of CCM with styrene demonstrated that saturation of the fatty acid side chains does not compromise the reactivity of the acrylic functionality, confirming the suitability of saturated POBMs for copolymerization with conventional vinyl monomers.

The thermal behavior and morphology of the resulting polymers were governed by the combined effects of fatty acid saturation and side chain length. Polymers containing predominantly C12 fatty acid side chains exhibited limited macromolecular ordering, whereas longer, highly saturated C16–C18 side chains promoted efficient packing and substantially more ordered polymer morphology. Poly(HG SBM) exhibited an enthalpy of fusion of 78 J g^−1^ and crystallinity degree of 37%, compared with 67 J g^−1^ and 26%, respectively, for poly(HG SBM-*co*-BBM). Within the polymer series investigated, semicrystalline morphologies were observed at degrees of unsaturation of approximately 4 mol% or lower, indicating that reduced structural irregularity of the fatty acid side chains, particularly when combined with longer side chains, facilitates the formation of ordered domains.

Overall, these results identify fatty acid saturation and side chain length as key molecular parameters for controlling polymerization, side chain packing, and macromolecular ordering in POBM-based polymeric materials. Importantly, semicrystalline morphology can be introduced through the intrinsic chemical structure of plant oil chosen for POBM synthesis.

This molecular design strategy expands the POBM platform beyond predominantly soft, amorphous polymers and provides a route toward fully biobased semicrystalline materials with tunable properties for biobased thermoplastics applications.

## Figures and Tables

**Table 1:** Fatty acid composition of oils used for POBM synthesis.

Fatty Acid Fragments, %	Oil		
	Babassu	Coconut	Hydrogenated Soybean
Linoleic * (18:2)	1.8	-	-
Oleic * (18:1)	10.2	4.5	2.2
Stearic (18:0)	1.8–7.4 [18]	2–4 [18]	87.2–91.1 [19,20]
Palmitic (16:0)	5.2–11 [18]	7.5–10.2 [18]	8.9–10 [19,20]
Myristic (14:0)	11–27 [18]	16.8–21 [18]	-
Lauric (12:0)	40–55 [18]	45.1–53.2 [18]	-
Capric (10:0)	1.2–7.6 [18]	5–8 [18]	-
Caprylic (8:0)	2.6–7.3 [18]	4.6–10 [18]	-

* Determined by ^1^H NMR (Figures S1–S3) according to the procedure [21].

**Table 2:** Structural and compositional characterization of POBM-based (co)polymers.

Sample	Monomer Feed, mol%	Experimental Composition, mol%	*U*, mol%	M_n_	Đ	Yield, %
poly(HG SBM)	HG SBM 100	HG SBM 100	2.2	74,800	3.6	- *
poly(HG SBM-*co*-BBM)	HG SBM:BBM 84:16	HG SBM:BBM 81:19	4	53,700	3.9	- *
poly(CCM)	CCM 100	CCM 100	4.5	39,700	5.5	65
poly(BBM-*co*-CCM)	BBM:CCM 70:30	BBM:CCM 54:46	8	44,500	5.9	64
poly(BBM)	BBM 100	BBM 100	12	42,000	5.4	80

* The hydrogenated soybean oil-based monomer exhibited limited solubility in methanol. Therefore, the yield could not be determined accurately, and the resulting (co)polymers were used as obtained.

## Data Availability

The original contributions presented in this study are included in the article/Supplementary Material. Further inquiries can be directed to the corresponding author.

## Supplementary Materials

The following supporting information can be downloaded at: https://www.mdpi.com/article/10.3390/molecules31203650/s1. Figure S1: ^1^H NMR spectrum of babassu oil; Figure S2: ^1^H NMR spectrum of coconut oil; Figure S3: ^1^H NMR spectrum of hydrogenated soybean oil; Figure S4: Mass spectrum of coconut (CCM) oil-based acrylic monomer; Figure S5: Mass spectrum of babassu (BBM) oil-based acrylic monomer; Figure S6: Mass spectrum of hydrogenated soybean (HG SBM) oil-based acrylic monomer; Figure S7: Conversion–time plots for CCM homopolymerization at different initiator concentrations; Figure S8: ^1^H NMR spectrum of poly(BBM); Figure S9: ^1^H NMR spectrum of poly(BBM-*co*-CCM); Figure S10: ^1^H NMR spectrum of poly(CCM); Figure S11: ^1^H NMR spectrum of poly(HG SBM-*co*-BBM); Figure S12: ^1^H NMR spectrum of poly(HG SBM); Figure S13: GPC chromatograms of poly(BBM), poly(BBM-*co*-CCM), poly(CCM), poly(HG SBM) and poly(HG SBM-*co*-BBM); Figure S14: DSC thermograms of (A) poly(BBM), (B) poly(BBM-*co*-CCM), (C) poly(CCM), (D) poly(HG SBM), (E) poly(HG SBM-*co*-BBM); Figure S15: MDSC thermograms of (A) poly(BBM), (B) poly(HG SBM), (C) poly(HG SBM-*co*-BBM).

## Abbreviations

POBMs—plant oil-based acrylic monomers; CCM—coconut oil-based acrylic monomer; BBM—babassu oil-based acrylic monomer; HG SBM—hydrogenated oil-based acrylic monomer; FTIR—Fourier Transform Infrared Spectroscopy; ^1^H NMR—Proton Nuclear Magnetic Resonance Spectroscopy; ESI-MS—Electrospray Ionization Mass Spectrometry.
