# Supplementary material for: Effect of Fatty Acid Saturation and Side-Chain Length on the Thermal Behavior and Morphology of Plant Oil-Based Acrylic Polymers
Source: Molecules. 2026 Oct 13;31(20):3650. doi: 10.3390/molecules31203650 (PMC13707671; doi:10.3390/molecules31203650)
Supplement: Supplementary file 1 [file molecules-31-03650-s001.zip › molecules-4563106-supplementary.pdf]

Supporting Information

Effect of Fatty Acid Saturation and Side-Chain Length on the Thermal Behavior and Morphology of Plant Oil-Based Acrylic Polymers

Mariia Usiichuk[1], Bohdan Domnich[1], Yuliia Romaniuk[2], Vasylyna Kirianchuk[1], Andriy Voronov[1]

[1]*Department of Coatings and Polymeric Materials, North Dakota State University, Fargo, ND, 58108*

[2]*Department of Organic Chemistry, Lviv Polytechnic National University, Lviv 79013, Ukraine*

Corresponding author: andriy.voronov@ndsu.edu

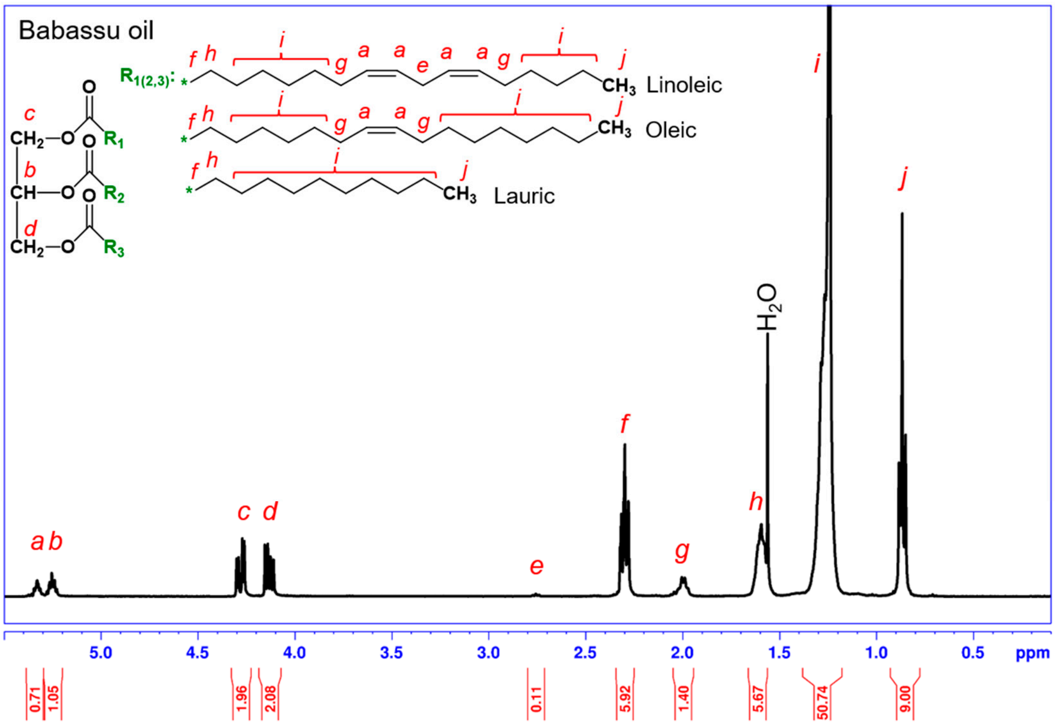

Figure S1. ^{1}H NMR spectrum of babassu oil.

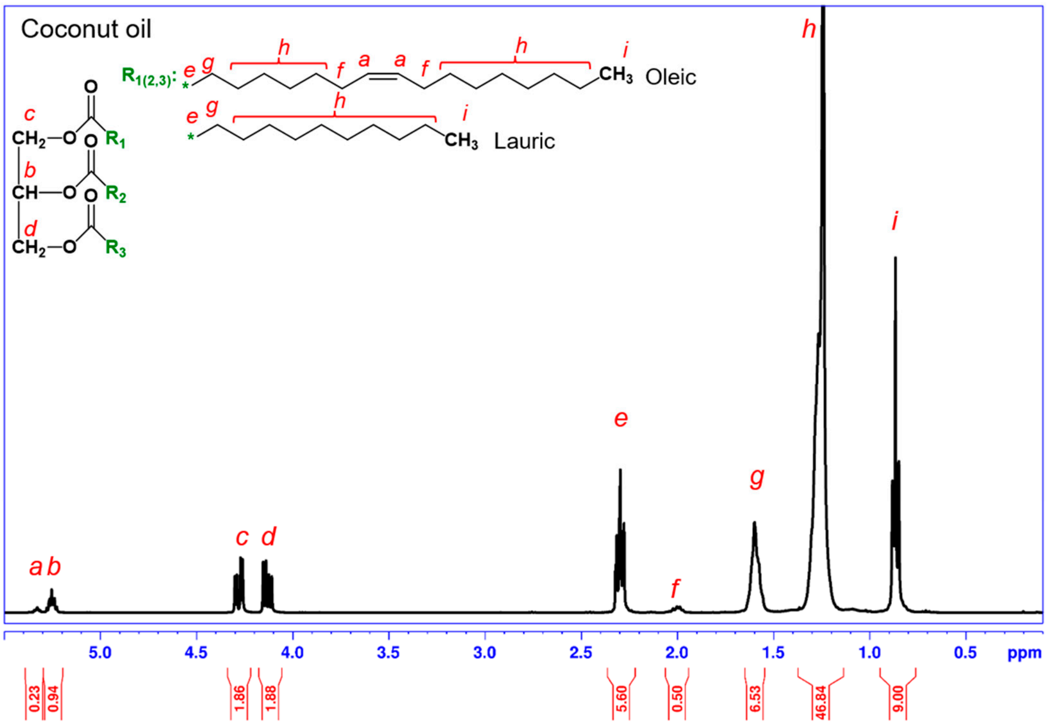

Figure S2. ^{1}H NMR spectrum of coconut oil.

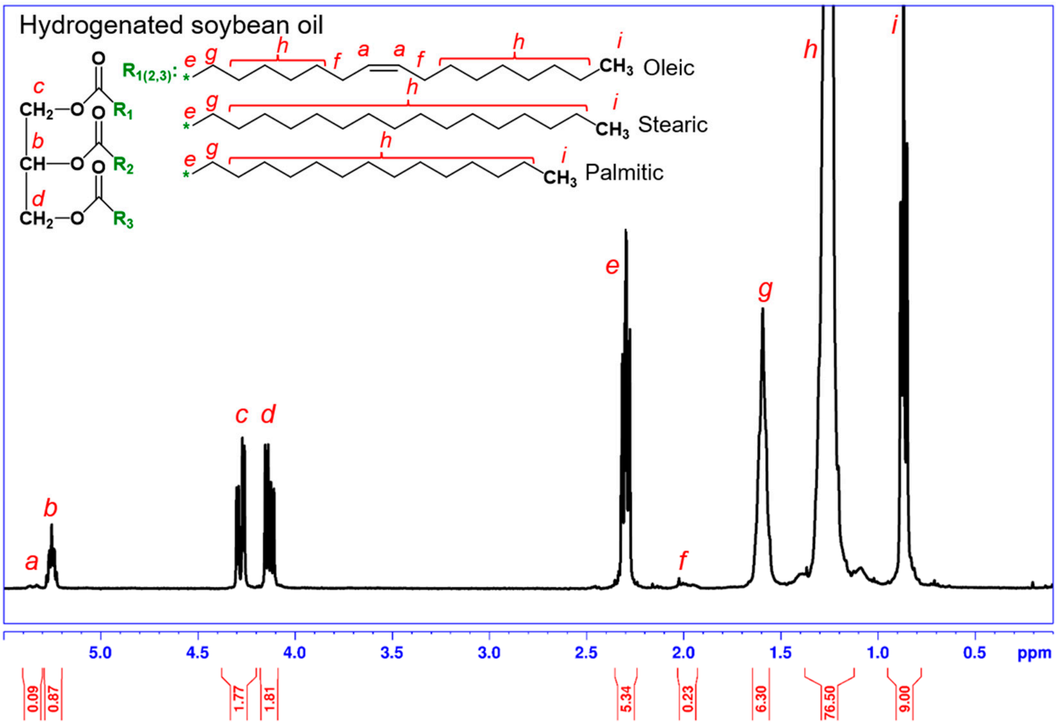

Figure S3. ^{1}H NMR spectrum of hydrogenated soybean oil.

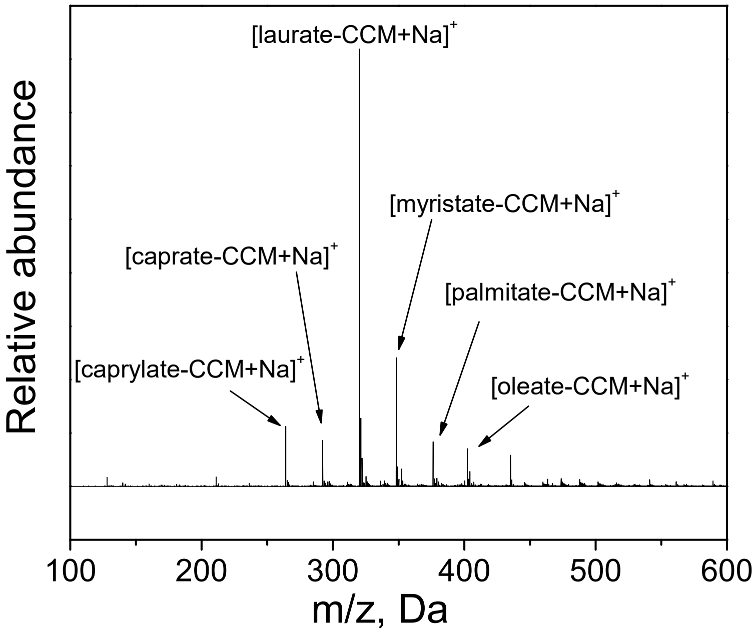

Figure S4. Mass spectrum of coconut (CCM) oil-based acrylic monomer.

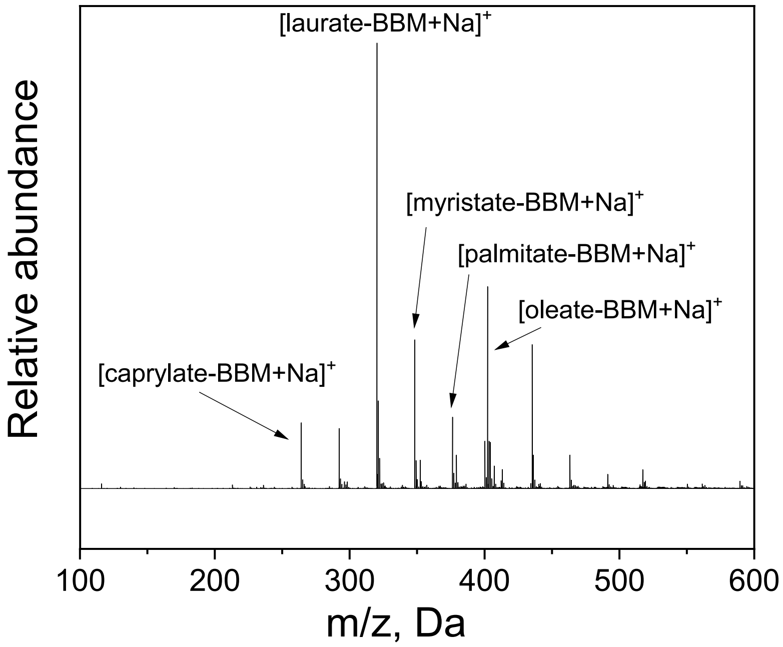

Figure S5. Mass spectrum of babassu (BBM) oil-based acrylic monomer.

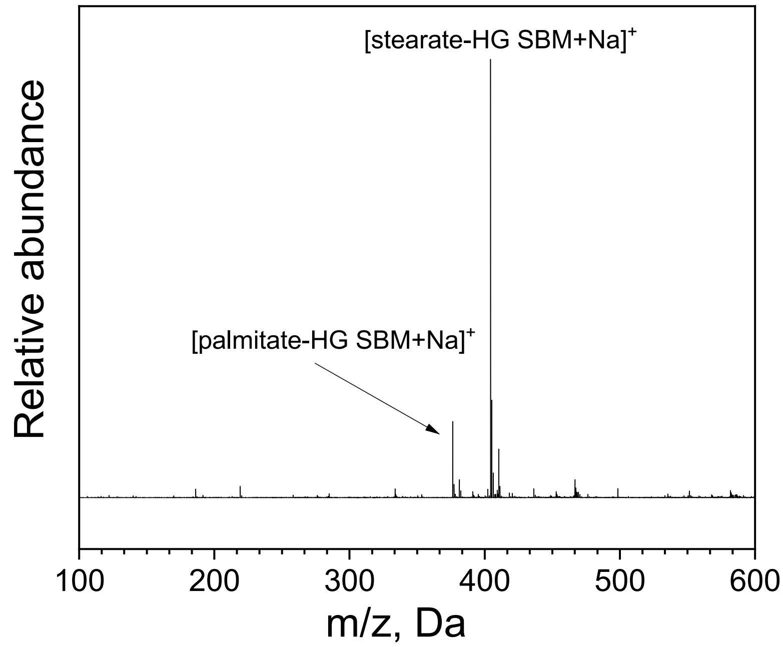

Figure S6. Mass spectrum of hydrogenated soybean (HG SBM) oil-based acrylic monomer.

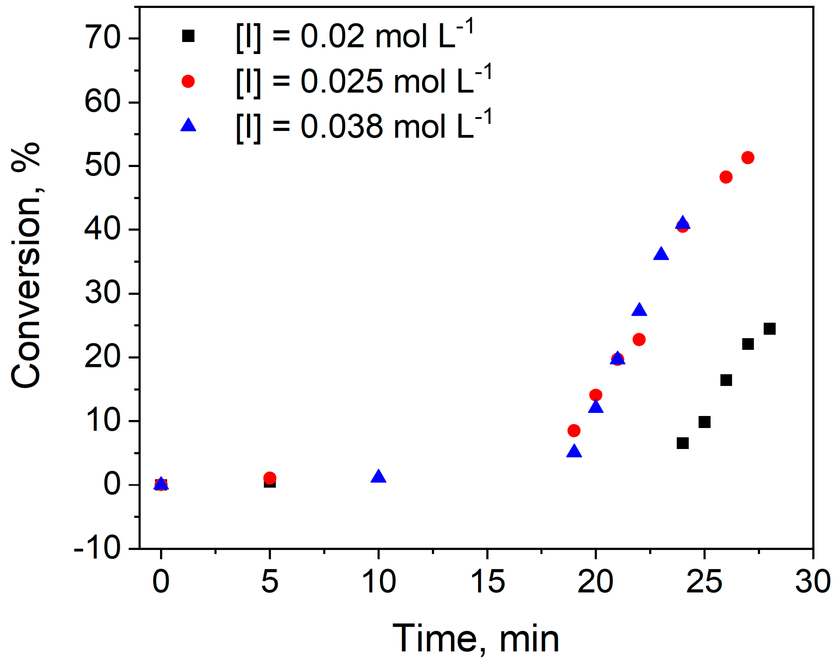

Figure S7. Conversion-time plots for CCM homopolymerization at different initiator concentrations.

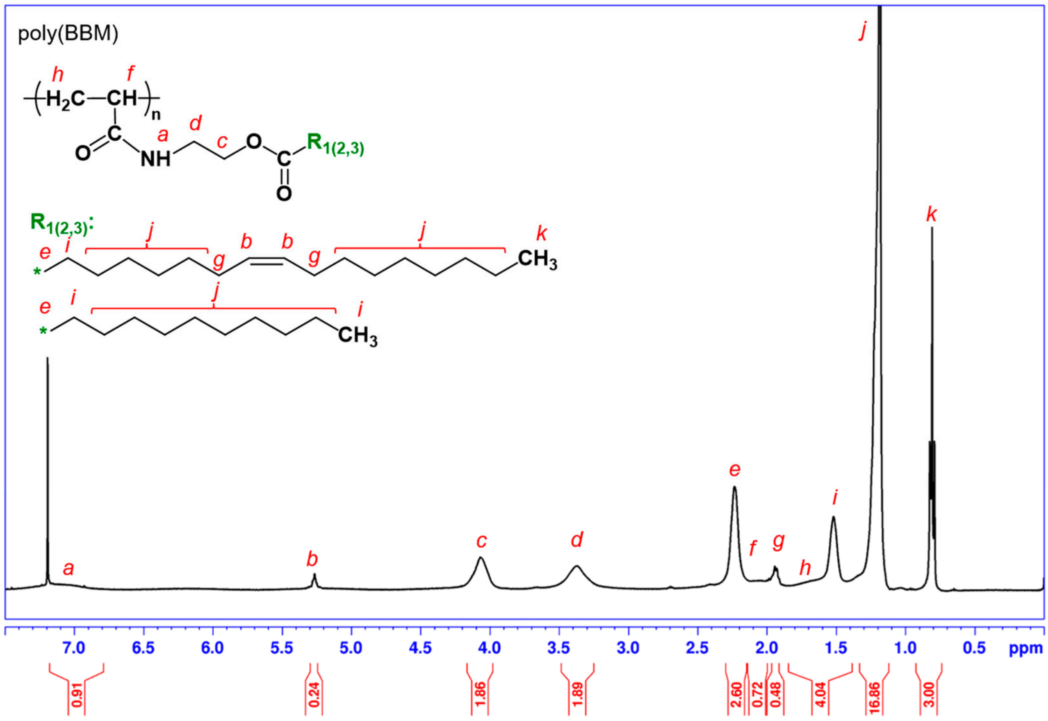

Figure S8. ^{1}H NMR spectrum of poly(BBM).

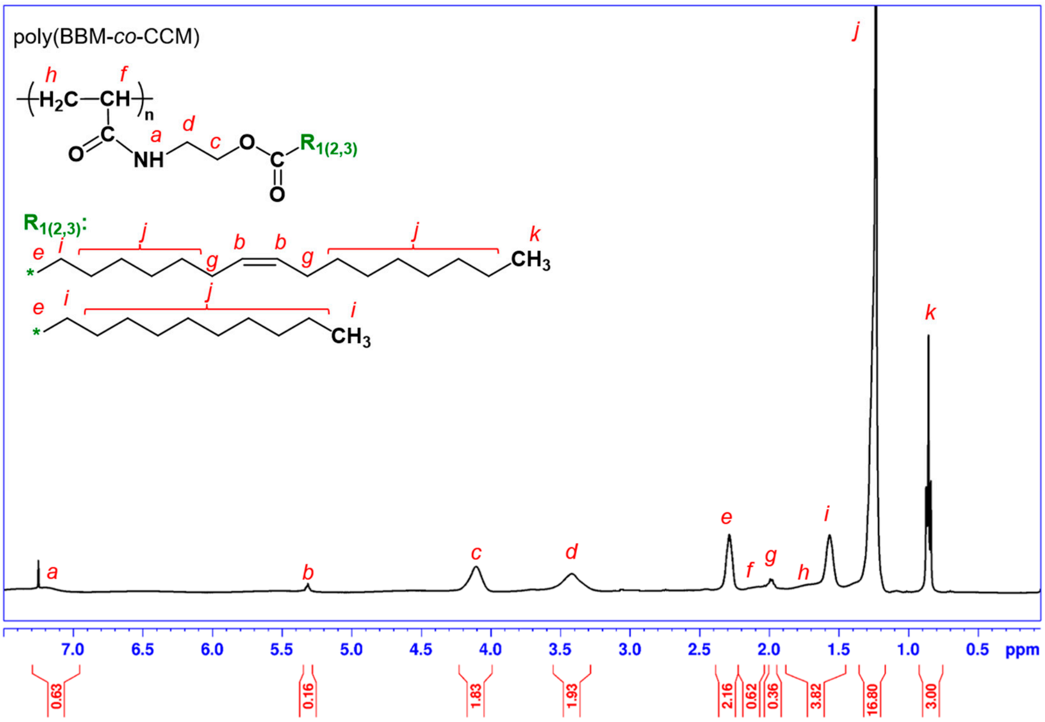

Figure S9. ^{1}H NMR spectrum of poly(BBM-*co*-CCM).

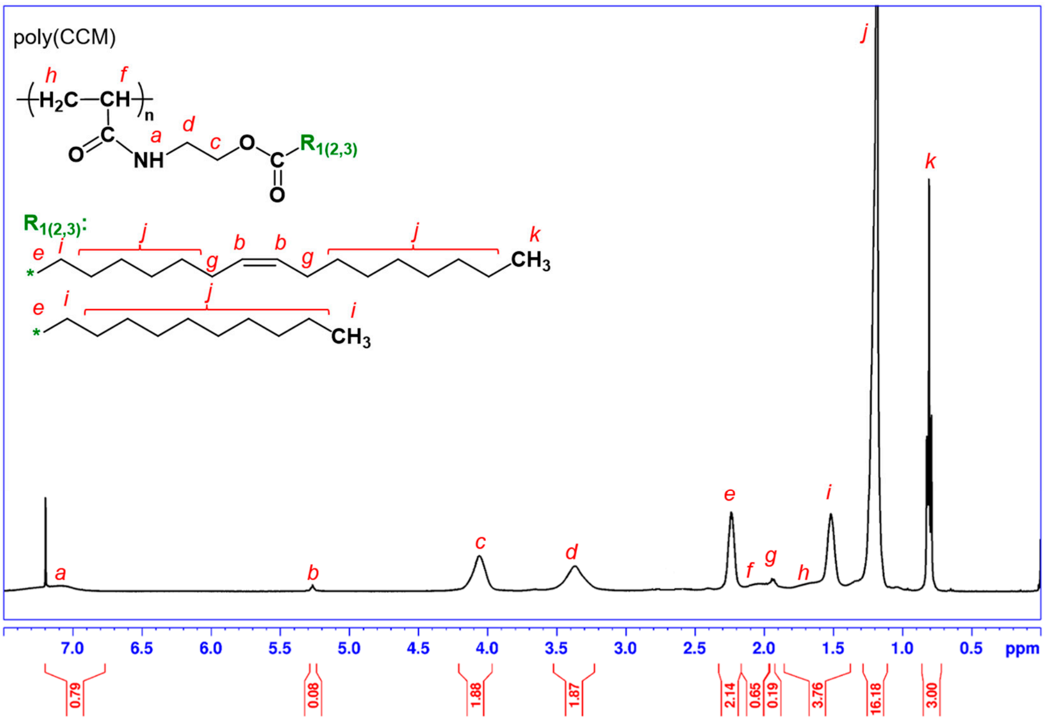

Figure S10. ^{1}H NMR spectrum of poly(CCM).

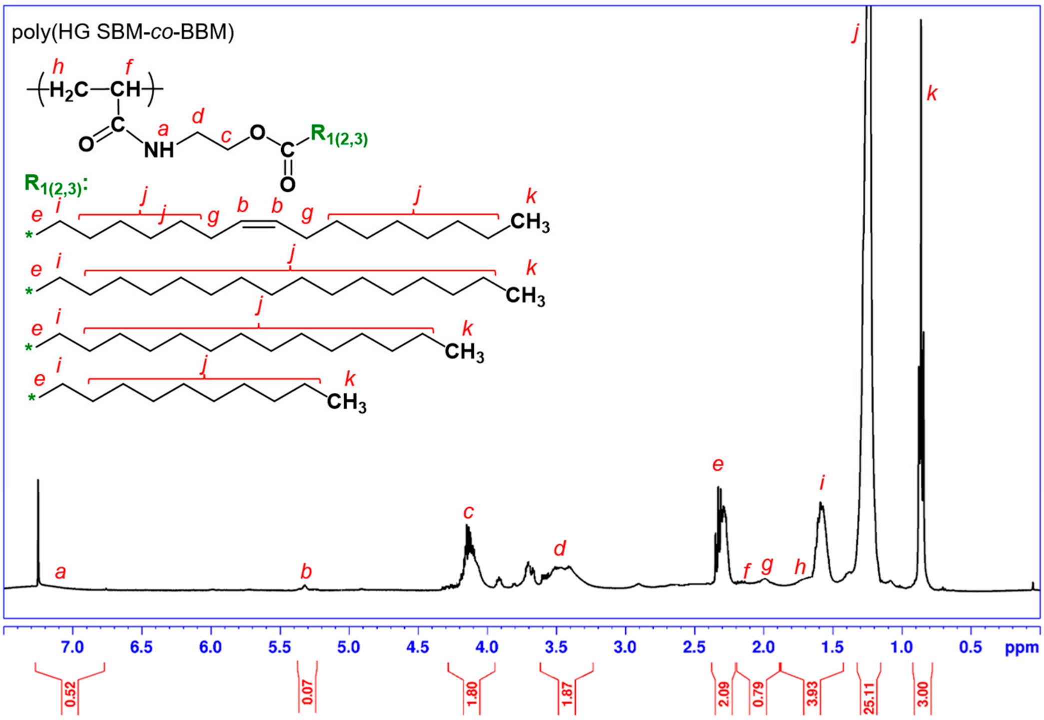

Figure S11. ^{1}H NMR spectrum of poly(HG SBM-*co*-BBM).

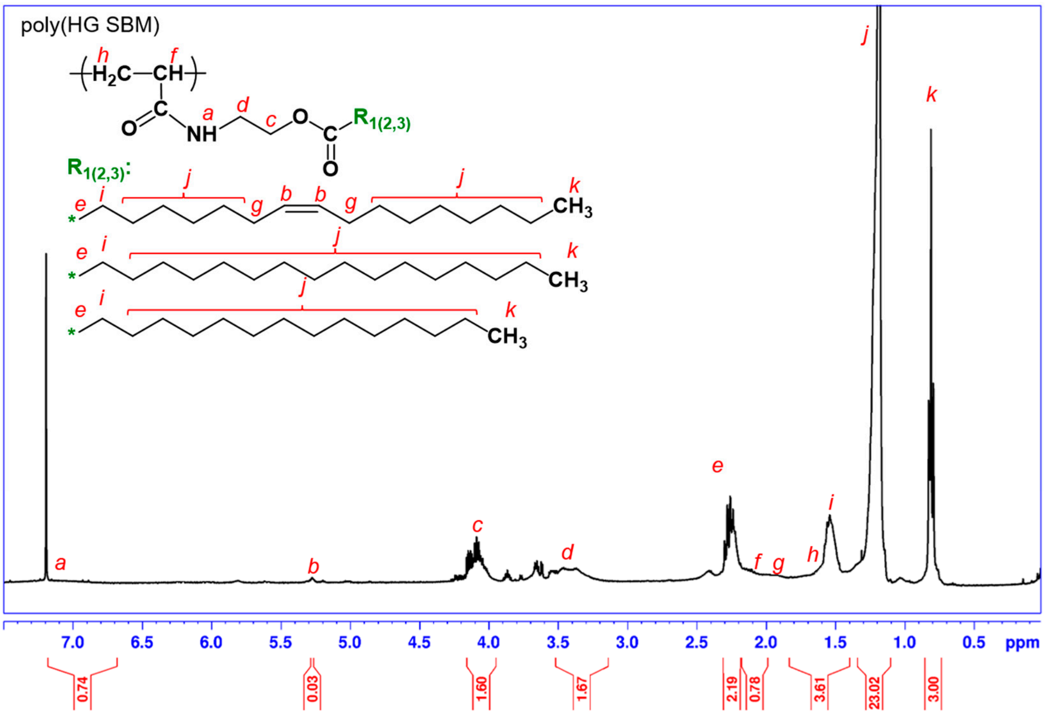

Figure S12. ^{1}H NMR spectrum of poly(HG SBM).

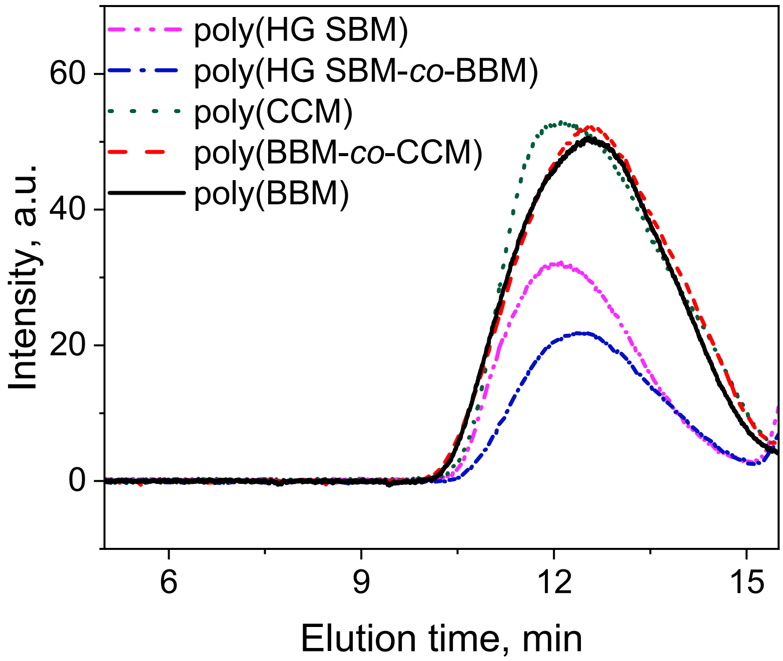

Figure S13. GPC chromatograms of poly(BBM), poly(BBM-*co*-CCM), poly(CCM), poly(HG SBM) and poly(HG SBM-*co*-BBM).

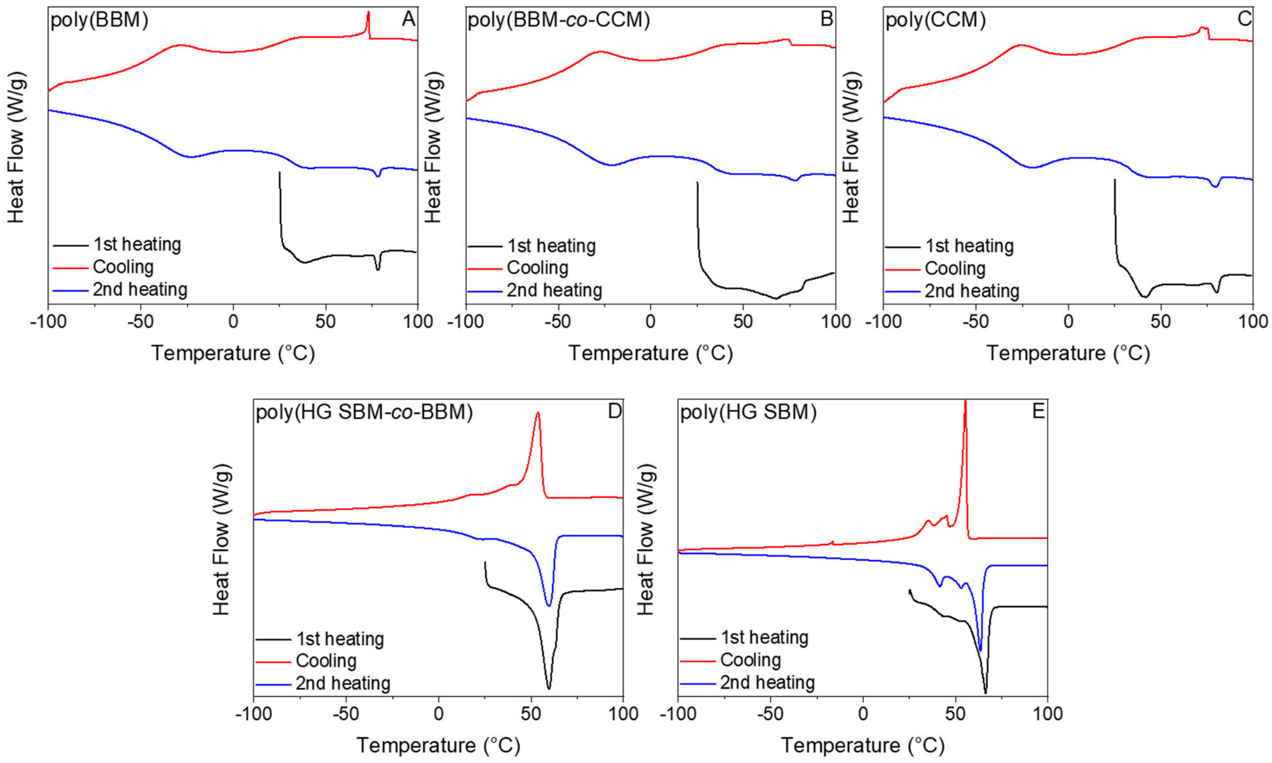

Figure S14. DSC thermograms of **(A)** poly(BBM), **(B)** poly(BBM-*co*-CCM), **(C)** poly(CCM), **(D)** poly(HG SBM), **(E)** poly(HG SBM-*co*-BBM).

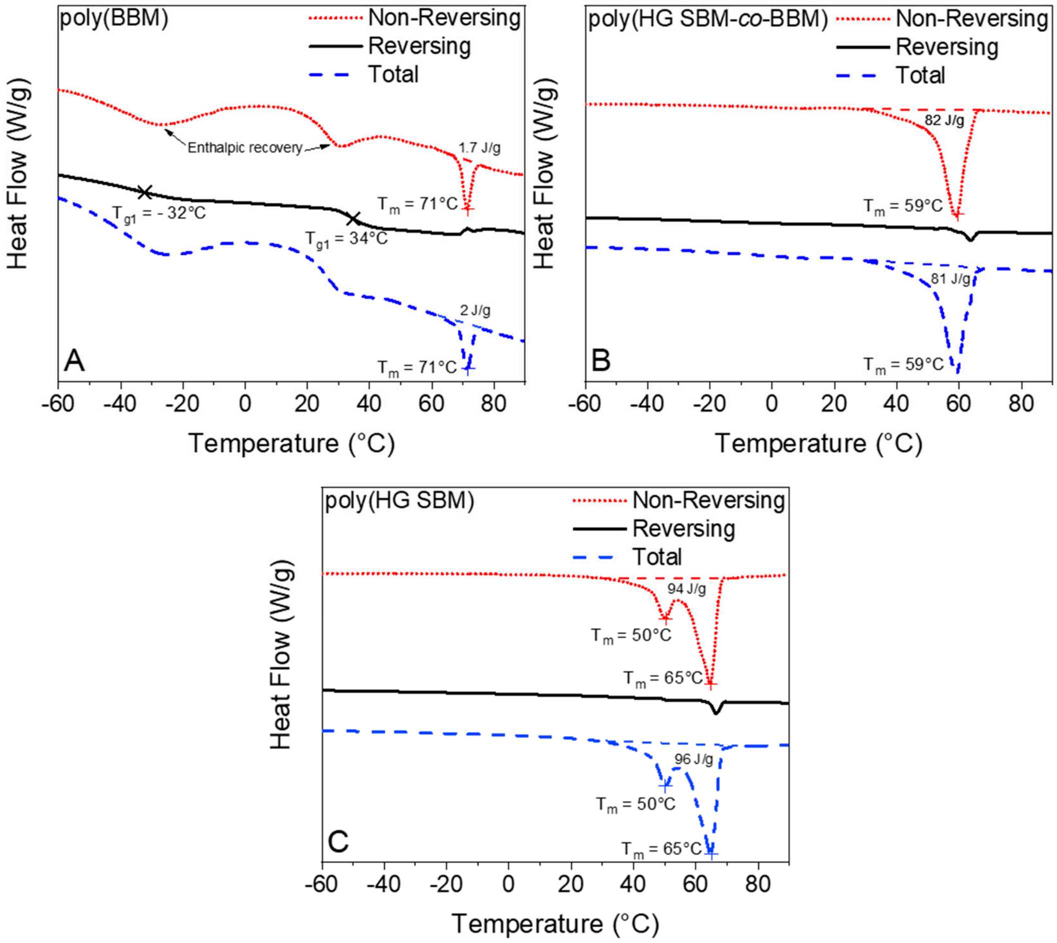

Figure S15. MDSC thermograms of **(A)** poly(BBM), **(B)** poly(HG SBM), **(C)** poly(HG SBM-*co*-BBM).
